# Gum Rosin as a Size Control Agent of Poly(Butylene Adipate-Co-Terephthalate) (PBAT) Domains to Increase the Toughness of Packaging Formulations Based on Polylactic Acid (PLA)

**DOI:** 10.3390/polym13121913

**Published:** 2021-06-08

**Authors:** Miguel Aldas, José Miguel Ferri, Dana Luca Motoc, Laura Peponi, Marina Patricia Arrieta, Juan López-Martínez

**Affiliations:** 1 Instituto de Tecnología de Materiales (ITM), Universitat Politècnica de València (UPV), 03801 Alcoy, Spain; jlopezm@mcm.upv.es; 2 Departamento de Ciencia de Alimentos y Biotecnología, Facultad de Ingeniería Química y Agroindustria, Escuela Politécnica Nacional, 170517 Quito, Ecuador; 3 Department of Automotive and Transport Engineering, Transilvania University of Brasov, Eroilor Av., 500036 Brasov, Romania; danaluca@unitbv.ro; 4 Instituto de Ciencia y Tecnología de Polímeros (ICTP-CSIC), C/Juan de la Cierva 3, 28006 Madrid, Spain; lpeponi@ictp.csic.es; 5 Departamento de Ingeniería Química Industrial y del Medio Ambiente, Escuela Técnica Superior de Ingenieros Industriales, Universidad Politécnica de Madrid (ETSII-UPM), Calle José Gutiérrez Abascal 2, 28006 Madrid, Spain; m.arrieta@upm.es; 6 Grupo de Investigación: Polímeros, Caracterización y Aplicaciones (POLCA), 28006 Madrid, Spain

**Keywords:** polylactic acid, PLA, poly(butylene adipate-co-terephthalate, PBAT, gum rosin, biodegradable polymers, barrier properties

## Abstract

Gum rosin (GR) was used as a natural additive to improve the compatibility between polylactic acid, PLA, and poly(butylene adipate-co-terephthalate, PBAT, blended with 20 wt.% of PBAT (PLA/PBAT). The PBAT was used as a soft component to increase the ductility of PLA and its fracture toughness. The coalescence of the PBAT domains was possible due to the plasticization effect of the GR component. These domains contributed to increasing the toughness of the final material due to the variation and control of the PBAT domains’ size and consequently, reducing the stress concentration points. The GR was used in contents of 5, 10, 15, and 20 phr. Consequently, the flexural properties were improved and the impact resistance increased up to 80% in PLA/PBAT_15GR with respect to the PLA/PBAT formulation. Field emission scanning electron microscope (FESEM) images allowed observing that the size of PBAT domains of 2–3 µm was optimal to reduce the impact stress. Differential scanning calorimetry (DSC) analysis showed a reduction of up to 8 °C on the PLA melting temperature and up to 5.3 °C of the PLA glass transition temperature in the PLA/PBAT_20GR formulation, which indicates an improvement in the processability of PLA. Finally, transparent films with improved oxygen barrier performance and increased hydrophobicity were obtained suggesting the potential interest of these blends for the food packaging industry.

## 1. Introduction

Polylactic acid (PLA) is one of the most widely consumed biodegradable and compostable polymers. In the field of medicine, it is used for its excellent compatibility with the human body [1]. However, its low toughness makes it necessary to modify it by incorporating additives to obtain a more ductile material, with better barrier properties, higher hydrophobicity, and higher stability to temperature and external agents (UV, humidity, etc.). Its modification allows greater applicability, there being several industrial sectors in which it can be used, among them agriculture [2], packaging [3,4], medical fields [5], 3D printing [6], textile fibers [7], and composites [8,9,10]. 

The PLA processing method with the more favorable industrial viability is its physical mixing or blending [11,12]. The modification of biodegradable polymers through physical blending with another biodegradable polymer shows many advantages since it offers the opportunity to create a new material with desired properties. Moreover, it is relatively simple and cost-effective to blend polymeric materials in the melt state, based on available processing technologies commonly used at the industrial level (i.e., extrusion, injection molding, film-forming, etc.) [13]. Many biodegradable polymer formulations have been obtained by blending polymeric matrices to modulate their mechanical, thermal, rheological, and morphological behavior. The literature refers to blends of PLA with other polymers or copolymers such as poly(ε-caprolactone) (PCL) [14], thermoplastic starch (TPS) [15], poly(butylene succinate) (PBS) [16], poly(butylene succinate-co-adipate) (PBSA) [17], poly(glycolic acid) (PGA) [18], poly(hydroxybutyrate) (PHB) [12,19], poly(hydroxybutyrate-co-valerate) (PHBV) [20], and poly(butylene adipate-co-terephthalate) (PBAT) [21], among others. Among these, PBAT has gained interest in the development of PLA/PBAT blends intended for film manufacturing due to its high flexibility [22] and its inherent biodegradable character [23]. However, in most of the scientific works reported up to now, poor miscibility or total immiscibility between components of the blend was observed and the expected synergism to improve the overall properties was not achieved.

Currently, additives can be used to increase the miscibility between polymers through various modification mechanisms that seek to increase either the interaction between the different polymeric phases through compatibilization, the plasticization of one of the components to increase the free volume, which facilitates the miscibility of a second component, or interaction through free radicals (reactive mixing). The components of the blend, together with the reactive agent, undergo a reaction and molecular chemical change that influence the mechanical and thermal properties. Wang et al. formulated PLA-based blends with different PBAT contents, additivated with 0.75% of Joncryl ADR 4368. As a result, the compatibility of both polymers was increased. Specifically, the elongation at break was increased by 18% and the impact absorption energy went from 4.85 kJ/m^2^ for the PLA/20PBAT to 5.21 kJ/m^2^ for the formulation 80PLA-20PBAT_0.75Joncryl [24]. Arruda et al. also used Joncryl ADR-4368 on PLA-PBAT blends and demonstrated its compatibilizing effect. The formulation 60PLA-40PBAT-0.6Joncryl increased the elongation at break by 1200% (compared to the percentage of elongation of neat PLA). Such a compatibilizer acts as a crosslinking and/or branching agent for both polymers, providing higher strength and Young’s modulus to each of the polymers separately, although it increases the ductility of all the studied formulations [25]. Wu et al. added 0.3, 0.5, and 0.75 wt.% of 2,5-bis (tert-butyl peroxy) -2,5-dimethyl hexane (Luperox 101), observing a crosslinking reaction and some interaction between the components (PBAT and PBS) of the ternary PLA-based blend. By adding 0.3 phr of Luperox 101 to the PLA/20PBAT/20PBS formulation, it was possible to increase by up to 10 times the impact energy absorbed, with respect to the polymeric matrix without additives, considerably reducing the glass transition temperature, T_g_, of PLA and increasing the elongation at break [26]. On the other hand, some natural plasticizers such as vegetable oils (VO), obtained from seeds, are presented as an effective alternative, sometimes acting as compatibilizers [27,28]. Their use in the packaging sector is of interest due to the high resistance of plasticizers to migration in food contact conditions [29,30], being able to increase the solubility of the blend components and therefore, act as a compatibilizer in polymeric blends. These can be chemically modified allowing greater interaction with the polymeric chains thanks to the added functional groups, reacting with polar groups such as hydroxyl, carboxylic, etc. [31,32]. According to Bocque et al., an efficient plasticizer has to be able to increase the molecular free volume and be endowed with ester groups (reactive functional groups that provide cohesion) and aromatic groups that increase its compatibilizing effect [33]. In this sense, Carbonell-Verdú et al. compatibilized PLA blended with 20 wt.% of PBAT by using cottonseed oil-based derivatives [22]. They observed that the low miscibility of PLA/PBAT could be improved by compatibilization with epoxidized cottonseed oil (ECSO) and maleinized cottonseed oil (MCSO). Moreover, both additives were able to considerably increase the elongation at break of the PLA/PBAT blend without compromising mechanical strength.

On the other hand, colophony or gum rosin (GR) and its derivatives have gained interest in the field of polymeric materials as highly versatile and multifunctional natural additives, both with synthetic plastic matrices and with biodegradable matrices [34,35,36]. For example, Arrieta et al. used a gum rosin ester as a natural viscosity increasing agent in a blend based on polyvinyl chloride (PVC) plasticized with epoxidized linseed oil (ELO). In that study, the gum rosin derivative showed good compatibility with the PVC synthetic matrix. Furthermore, it was verified that a composition between 40 and 50 phr of rosin ester present in PVC can increase the viscosity of the blends up to 10 times [37]. On the other hand, in previous work, the effect of GR and two gum rosin esters on the properties of a commercial blend of TPS, PBAT, and PCL was studied, and the versatility of the resin and its derivatives were verified. Furthermore, the GR acted as a plasticizer, and on the other hand, the gum rosin esters provided a solubilizing and compatibilizing effect of the biodegradable blends. This behavior influenced the properties of each of the studied formulations, especially in those with 15 wt.% of GR where the processing temperature was reduced by up to 50 °C and the toughness increased up to 500%, compared to the neat polymeric matrix [38]. Finally, when the interaction between gum rosin and gum rosin derivatives with Mater-Bi type bioplastic was studied through microscopic techniques, the improvement of the miscibility of the components and the solubility effect conferred to the PBAT phase thanks to the compatibilizing effect of the GR and its derivatives were confirmed [39,40].

The main objective of this work is to compatibilize PLA/PBAT-based blends with GR and to study the plasticizing effect conferred by the GR on the PLA/PBAT binary blends, focusing on the use of a natural resin as the main novelty of the present work. The processability aspects, as well as the main mechanical properties of the formulations, the thermal stability, and the barrier properties were assessed. In addition, the microstructure was studied by field emission scanning electron microscopy (FESEM) which allowed to see the interface of both polymers and to evaluate the plasticizing effect of GR on the toughness of the PLA/PBAT blend.

## 2. Materials and Methods

### 2.1. Materials

The poly(lactic acid) (PLA) used was Ingeo^TM^ Biopolymer, commercial-grade 6201D, supplied by NatureWorks LLC (Minnetonka, MN, USA). The pellet’s density was 1.24 g/cm^3^, the melt flow index was 15–30 g/10 min measured at 210 °C, and it contained 2% of D-lactic acid. The poly(butylene adipate-co-terephthalate) (PBAT) was a commercial-grade Biocosafe^TM^ 2003 F, supplied by Xinfu Pharmaceutical Co. Ltd. (Zhejiang, China) and it was characterized by a density of 1.25 g/cm^3^ and a melt flow index < 6 g/10 min at 190 °C. As the additive, gum rosin (GR), supplied by Sigma-Aldrich (Mostoles, Spain), was used.

### 2.2. Blends Preparation

Blends containing PLA, PBAT, and GR were prepared with different compositions, which are summarized in Table 1. The percentage of PBAT in the PLA matrix was fixed at 20 wt.% based on results reported in the literature [22]. At this percentage, PLA-20%PBAT blends have given interesting results in terms of toughness. To study the effect of the resin in the PLA/PBAT blends, the content of gum rosin added was set from 0 to 20 phr (parts of GR per hundred parts of PLA/PBAT blend). Neat PLA, neat PBAT, and PBAT with 10 phr of GR were also prepared to compare their properties with the previous blends.

All materials were dried at 50 °C for 48 h in an air circulation oven before processing. After, the formulations were premixed in a zipper bag. To obtain the final materials, the procedure was followed as described: (1) extrusion of the material formulations in a twin-screw extruder (Dupra S.L, Castalla, Spain), L/D ratio of 25, with a temperature profile from 185 to 140 °C (from die to hopper) at 50 rpm; (2) milling into pellets; and (3) injection molding in an injection molding machine (Sprinter-11, Erinca S,L., Barcelona, Spain), with a temperature profile from 185 to 175 °C, to obtain test specimens. The test specimens were standard rectangular specimens (80 × 10 × 4 mm) and standard tensile specimens “1BA” (length ≥ 75 mm, width 10 mm, and thickness ≥ 2 mm) according to ISO 527 [41]. The films of each formulation were further obtained by the solvent casting method, using chloroform as solvent. In particular, 20 g of each formulation was dissolved in 100 mL of chloroform and heated at 30 °C under vigorous stirring for 1 h. The obtained solutions were cast onto a 20-cm diameter mold and the films were obtained after complete solvent evaporation at room temperature after 24 h.

### 2.3. Material Characterization

#### 2.3.1. Colorimetric Properties and Visual Appearance Evaluation

The color properties of each formulation were determined in the CIEL*a*b* color space using a Konica CM-3600d ColorFlex-Diff2, HunterLab, Hunter Associates Laboratory, Inc, (Reston, VA, USA), using the standard rectangular specimens as the sample. The instrument was calibrated with a white standard tile and the color coordinates: L* (lightness), a* (red-green) and b* (yellow-blue), and yellowness index (YI) were measured. Measurements were carried out in quintuplicate at random positions over the sample surface. For the visual appearance evaluation, film samples were placed on a background image, to evaluate their transparency and the change in tonality due to the incorporation of GR. All samples were photographed in the same ambient light conditions and the evaluated films presented a uniform and constant thickness of 200 μm.

#### 2.3.2. Mechanical Characterization

Tensile and flexural tests were carried out in a universal testing machine ELIB 30 from S.A.E. Ibertest (Madrid, Spain) at room temperature, according to ISO 527 [42] and ISO 178 [43], respectively. Both tests were performed with a loading cell of 5 kN and a test speed of 10 mm/min, using five samples from each formulation in each test. Moreover, the typical stress-strain curve for each formulation was plotted from one representative curve, which showed the average behavior of the formulation. Furthermore, the toughness of the materials was calculated with the area under this curve using the OriginPro2015 program. Five values of toughness were assessed for each formulation and the average and standard deviation values are reported.

Impact absorbed energy measurements were carried out with a Charpy pendulum machine from Metrotec S.A. (San Sebastian, Spain), using a 6 J pendulum under the ISO 179 [44]. Five specimens were tested, and the mean and standard deviation are reported.

The hardness of PLA/PBAT/GR formulations was measured using a Shore D durometer, Model 673-D, from Instrument J.Bot S.A. (Barcelona, Spain), following the guidelines of ISO 868 [45]. Twenty measurements were taken from aleatory parts of the samples, and the mean and standard deviation are reported as hardness values.

The heat deflection temperature (HDT) was determined by the A method according to ISO 75 [46], which recommends a load of 1.8 MPa and a heating rate of 120 °C/h. In addition, the Vicat softening temperature (VST) was assessed using the ISO 306 [47], method B (with a load of 50 N and a heating rate of 50 °C/h). Both tests were carried out in a VICAT/HDT station DEFLEX 687-A2 from Metrotec SA (San Sebastián, Spain). For each property, three specimens were tested, and the mean and standard deviation are reported.

#### 2.3.3. Microstructural Characterization

A field emission scanning electron microscope (FESEM) model Zeiss Ultra, from Oxford Instruments (Abingdon, UK) was used to obtain micrographs from the cryofractured surfaces of the rectangular samples. The acceleration voltage was set to 2 kV. Previously, the samples were coated with an ultrathin platinum layer in a vacuum using a coater, model MED020 (Leica, Leica Microsystems). Micrographs of PLA with GR have not been reported, since there is literature that shows the effect of gum rosin on the PLA matrix [48].

#### 2.3.4. Thermal and Thermomechanical Characterization 

The thermal properties of the PLA/PBAT/GR formulations were obtained by using two analyses, differential scanning calorimetry (DSC) (Mettler-Toledo model 821, Schwerzenbach, Switzerland) and thermogravimetric analysis (TGA) (Linseis, model TGA 1000, Linseis Messgeraete GmbH, Selb, Germany). In the programming of the DSC system, the thermal cycles were carried out, under a nitrogen atmosphere, with a heating and cooling rate of 10 °C/min. The purpose of the first heating was to eliminate the thermal history and was carried out from 25 °C to 200 °C. Later it was cooled down to −50 °C and finally, the second heating was carried out to 250 °C. The degree of crystallinity (X_c_), obtained from the DSC thermograms, was calculated using Equation (1).

(1) $$X_{c}\;\left(\%\right)=100\;\times \;\frac{\Delta H_{m}-\Delta H_{\mathrm{cc}}}{\Delta H_{m}\left(100\%\right)}\times \frac{1}{W_{\mathrm{PLA}}},$$

where ΔH_m_ and ΔH_cc_ are the melting and the cold crystallization enthalpies, respectively, ΔH_m_ (100%) is the calculated melting enthalpy of purely crystalline PLA (93 J/g) [49]. W_PLA_ is the weight fraction of PLA in the formulation.

The mass loss obtained by TGA was carried out at a heating rate of 10 °C/min, measured in the range of 30 °C to 800 °C, under a nitrogen atmosphere and with a flow rate of 30 mL/min. The T_5%_ was taken at the temperature where the 5% of mass loss was reached. The T_max_, the temperature where the degradation rate was maximum, was obtained at the peak of the first derivative of the TGA curve (DTG curves). Additionally, dynamic mechanical thermal analysis (DMTA) was performed in torsion mode, from −90 °C to 120 °C at a heating rate of 2 °C/min, a frequency of 1 Hz, and 0.1% of maximum deformation. The test was done on rectangular samples sizing 40 × 10 × 4 mm^3^ in an oscillatory rheometer AR G2 from TA Instruments (New Castle, DE, USA) equipped with a special clamp system for solid samples.

#### 2.3.5. Oxygen Permeability Measurements of PLA/PBAT/GR Formulations

Oxygen transition rate (OTR) values were obtained using an oxygen permeation analyzer from Systech Instruments-Model 8500 (Metrotec S.A, Lezo, Spain) at a pressure of 2.5 atm and room temperature. Films were introduced in the diffusion chamber. Pure oxygen (99.9% purity) flowed through the upper half of the sample chamber and nitrogen flowed through the lower half of the chamber. It dragged the oxygen flowing through the film and was measured by an oxygen detector. To obtain an average value of OTR per film thickness (OTR.e), three measurements were made. Thickness was measured precisely at 25 °C using a Digimatic Micrometer Series 293 MDC-Lite (Mitutoyo, Kanagawa, Japan) with an error of 0.001 mm. Twenty readings were taken at random positions over the 14 cm diameter circle films.

#### 2.3.6. Static Water Contact Angle Measurements of PLA/PBAT/GR Formulations

The wettability was measured by water contact angle at room temperature using an Easy Drop Standard goniometer FM140 (KRÜSS GmbH, Hamburg, Germany). The equipment was provided with a camera and analyzer software (Drop Shape Analysis SW21; DSA1 from KRÜSS GmbH, Hamburg, Germany). Ten contact angles were measured randomly using distiller water as contact liquid on the surface film with a microsyringe. Five measurements were carried out for each drop and the average value was calculated.

#### 2.3.7. Statistical Analysis

The statistical analysis was performed to establish the effect of the GR content on the properties of the PLA/PBAT matrix. The significant differences in all the properties were statistically assessed at 95% confidence level according to Tukey’s test using a one-way analysis of variance (ANOVA) by means of OriginPro2018 software (OriginLab, Northampton, MA, USA).

## 3. Results and Discussion

### 3.1. Visual Appearance and Color Properties 

**Figure 1:**
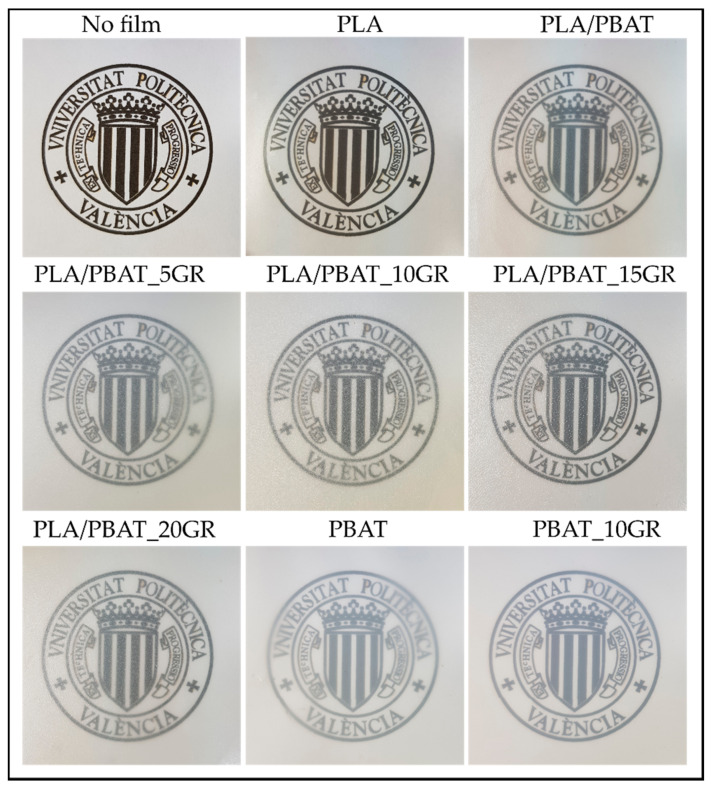
The visual appearance of PLA, PBAT, PBAT_10GR, PLA/PBAT, and PLA/PBAT with 5, 10, 15, and 20 phr GR resin films.

One of the most important requirements of packaging materials for consumers’ acceptance is seeing the packed food through the packaging material. Figure 1 shows the visual appearance of the obtained films. It is possible to observe the high transparency of the films suggesting their potential application in the food packaging field. The addition of 20 wt.% of PBAT showed some loss in transparency. The further incorporation of GR also led to a partial decrease in the high transparency of PLA, which was more marked with the increasing amount of GR. In the case of the neat PBAT formulation, it showed less transparency than neat PLA and the incorporation of GR was practically imperceptible on the transparency of the sample (PBAT_10GR).

The film color properties were determined in the CIEL*a*b* space and the results are summarized in Table 2. PLA showed the highest lightness (L*), in good accordance with the visual appearance of the films. Meanwhile, the lowest lightness was observed for the PLA/PBAT blend with the higher amount of GR (PLA/PBAT_20GR). The a* values (which correspond to red-green coloration), although significant (*p* < 0.05), did not highly change its values in any of the studied formulations. In contrast, the b* coordinate and the yellowness index (YI) significantly (*p* < 0.05) and considerably increased with the resin content, since the blend became more yellow due to the inherent characteristics of the GR. 

### 3.2. Microstructural Characterization

**Figure 2:**
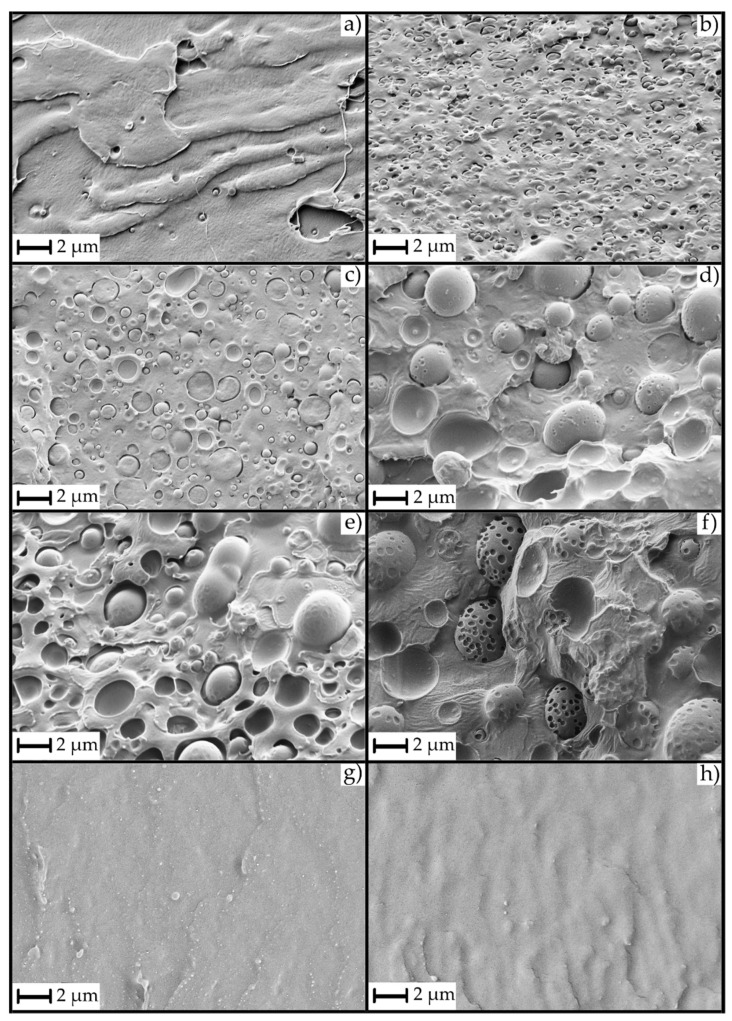
FESEM images at 5000 X of studied materials: (**a**) PLA, (**b**) PLA/PBAT, (**c**) PLA/PBAT_5GR, (**d**) PLA/PBAT_10GR, (**e**) PLA/PBAT_15GR, (**f**) PLA/PBAT_20GR, (**g**) PBAT, and (**h**) PBAT_10GR.

Figure 2 shows the effect of the GR resin on the partially miscible PLA/PBAT blends, as well as the effect on the neat PBAT, taken as reference. Neat PLA (Figure 2a) showed a flat surface with small prominences, characteristic of a brittle break of the material under cryofracture conditions. The blend of PLA with 20% PBAT (PLA/PBAT, Figure 2b) showed a smoother surface with PBAT domains sizing less than 0.5 µm, a characteristic size that shows that the components have partial miscibility, although it is very poor [22]. The incorporation of a 5 phr of GR (Figure 2c) showed significant differences in the morphology of the cryofracture surface. Specifically, the PBAT domains were larger (between 0.5–1.5 µm), with an average size of 1 µm. Moreover, PBAT domains turned from presenting irregular shapes (Figure 2b) to almost perfect spherical shapes. This is indicative of the loss of affinity and miscibility between PLA and PBAT when adding GR. However, with a 5 phr of GR, there was still some interaction between the PLA and the PBAT matrices since the PBAT domains broke through the plane of the fracture (in other words, the PBAT spheres broke through the crack of the fracture). This means that the PLA-PBAT interaction was greater than the cohesion forces of PBAT. Moreover, it should be highlighted that, although higher, the PBAT domains showed good adhesion with the PLA matrix at the interface.

This type of fracture did not persist with higher concentrations of GR. Figure 2d,e show the PLA/PBAT formulation with 10 and 15 phr of GR, respectively. In these images, the larger size of the PBAT domains (1.5–4 µm) can be observed. Although there were slight differences in PBAT domain sizes, their fractures were not similar to Figure 2c. In fact, in these formulations, the PBAT domains were not broken and showed complete PBAT spheres with small (nanoscale) domains of GR. The non-breakage of the PBAT spheres was due to the lower miscibility and interaction between the PLA and the PBAT domains, which is due to the phobic effect that exists between the PLA matrix and the GR resin [48]. This lack of interaction generated points of zero interaction around the PBAT spheres that prevented their breakage, although it improved the impact energy absorption, probably due to the PBAT domains still showing good adhesion with the PLA matrix at the interface, since there was not a gap between both polymeric matrices. Finally, Figure 2f shows the formulation with a 20 phr of GR, where the PBAT domains had an approximate average size of 4–5 µm and exhibited signs of GR saturation within them. These nanodomains, which were less evident in the other formulations, generated an important reduction in the PLA/PBAT interactions and resulted in a decrease of the impact energy absorption. Therefore, the formulations with PBAT domain sizes of 2–3 µm were those that improved the impact energy absorption or the toughness. 

Additionally, Figure 2g,h show the cryofractured surface of neat PBAT and PBAT with 10 phr of GR. It is possible to verify, along with the mechanical properties, that GR acted as a plasticizer for the PBAT. There were no appreciable differences in the morphology of the cryofractured surfaces of both materials since PBAT is a soft material that became even more softer by the addition of GR, demonstrating the plasticization effect of the GR.

### 3.3. Mechanical Properties of the PLA/PBAT/GR Formulations

Table 3 shows the main values of the mechanical properties, maximum tensile and flexural strength, Young’s moduli, elongation at break, impact absorption energy (Charpy), Shore D hardness, and the HDT temperature of each obtained formulation, as well as the neat PLA as a reference. 

**Figure 3:**
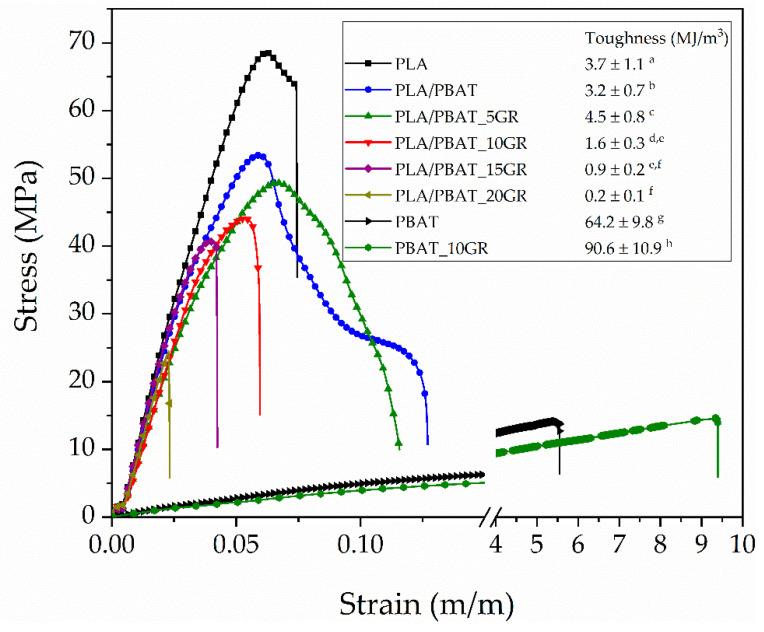
The toughness of PLA, PBAT, PBAT_10GR, PLA/PBAT, and PLA/PBAT with 5, 10, 15, and 20 phr of GR resin. ^a–h^ Different letters show statistically significant differences between formulations (*p* < 0.05).

It was observed that the tensile strength of the PLA/PBAT formulations significantly decreased (*p* < 0.05) by 6.3%, 17%, 23.2%, and 29.1% (compared to PLA/PBAT) when adding 5, 10, 15, and 20 phr of GR, respectively. However, the toughness (calculated as the area under the stress-strain curve) of the formulation with a 5 phr of GR (PLA/PBAT_5GR) significantly increased (*p* < 0.05) compared to the base formulation PLA/PBAT by 40%, as shown in Figure 3. In Figure 3, it is observed that as the content of GR increased, the toughness (energy per unit volume) of the formulations significantly decreased (*p* < 0.05). In contrast, the drastic increase in the toughness of PBAT by adding 10 phr of GR should be noted. This fact explains that GR acts as a PBAT plasticizer since it increased its elongation and decreased its tensile strength.

On the other hand, it was observed that when adding 5 and 10 phr of GR, Young’s modulus (Table 3) did not significantly decrease (*p* > 0.05) compared to PLA/PBAT. However, from contents higher than 10 phr of GR, where GR saturates notoriously in the PBAT domains (as shown by FESEM in Figure 2), the saturations behaved as reinforcements, increasing Young’s modulus mean values. Specifically, Young’s modulus of the formulations with a 15 phr GR showed a reduction of less than 10% relative to the reference formulation (PLA/PBAT). For contents of 20 phr GR, it showed an increase of the value, being 6.6% higher than the reference. The tendency of the mean values suggests that at low contents, GR acts as a plasticizer of the PBAT component, making the PBAT domains less compatible with the PLA matrix (because PLA is incompatible with GR). At contents higher than the saturation point (at 10 phr of GR), some nano-scale domains were generated. These domains acted as reinforcement, increasing the mean value of the Young’s modulus of the materials. It should be noted that GR resin has higher affinity and therefore higher compatibility with PBAT, confirming its ability to plasticize it.

Table 3 also shows the mechanical properties of PBAT and PBAT_10GR, the behavior of which can be considered as an indicator of the compatibility between the PBAT and the GR. The significant reduction (*p* < 0.05) in Shore D hardness, which went from 41 to 38 and the reduction in Young’s modulus, both in the flexion and in the tensile test, of the PBAT_10GR formulation compared to neat PBAT, show that the GR exerted a plasticizing effect on the PBAT matrix. Moreover, a significant increase (*p* < 0.05) of 47.6% in elongation was observed, which reinforces the idea of the plasticizing effect. However, the elongation at break of the PLA/PBAT formulations with different GR contents suffered a significant reduction (*p* < 0.05) of 55.5%, 68.3%, 76.8%, and 88.0%, compared to the PLA/PBAT formulation, when adding 5, 10, 15, and 20 phr of GR, respectively. It is well known that the miscibility of PBAT and PLA is partial with some compatibility [22,24,50]. Nevertheless, a phenomenon of coalescence of the PBAT domains was generated with the addition of GR to the PLA/PBAT formulation, creating larger domains and reducing the interaction between PLA and PBAT. This behavior can be explained since the PLA does not assimilate the GR in its matrix, which remains isolated. This phenomenon makes the GR assimilation by the PBAT domains easier, and therefore, the size of these domains increased with the GR content.

In contrast, a significant increase (*p* < 0.05) in the impact energy absorption was observed (Table 3) when incorporating 10 and 15 phr GR into the PLA/PBAT formulation, managing to increase it by 75.5 and 79.2%, respectively. For contents higher than 15 phr GR, the impact energy absorption mean values began to decrease due to saturation of the GR on the PBAT domains, generating a phase separation between the PLA/PBAT matrix and the GR saturations. This effect was corroborated by FESEM analysis (Figure 2f). According to the literature, when the domains of the ductile and dispersed material in a rigid polymeric matrix have a size of 2–5 µm, the energy absorption is maximum. This phenomenon has been demonstrated in a large number of materials, for example in the synthesis of HIPS [51], where polybutadiene forms domains into the PS matrix. At smaller or larger domain sizes, the energy absorption value decreases again. Therefore, the stress concentration generated in materials with poor interaction will depend on the size of the domains of the minority component, in this case, the PBAT-GR system. In the present study, the toughness of materials, especially at impact, could be controlled and improved thanks to the phobicity between PLA and GR and the affinity between PBAT and GR. If the GR resin showed a good affinity with both polymers (PLA and PBAT), the toughness modification would depend exclusively on the composition of the polymeric components and their interaction.

Finally, the hardness (Shore D) of the studied blends significantly varied (*p* < 0.05) with the incorporation of GR. This property significantly increased for 15 and 20 phr of GR contents. The plasticizing effect of the GR resin on the neat PBAT significantly reduced the hardness (Table 3). The HDT significantly decreased (*p* < 0.05) with increasing GR content, going from 57.8 °C for PLA/PBAT to 53.8 °C for the formulation with a 20 phr of GR. Therefore, the processability of these materials improved as the GR content increased.

### 3.4. Thermal and Thermomechanical Properties of the PLA/PBAT/GR Formulations

**Figure 4:**
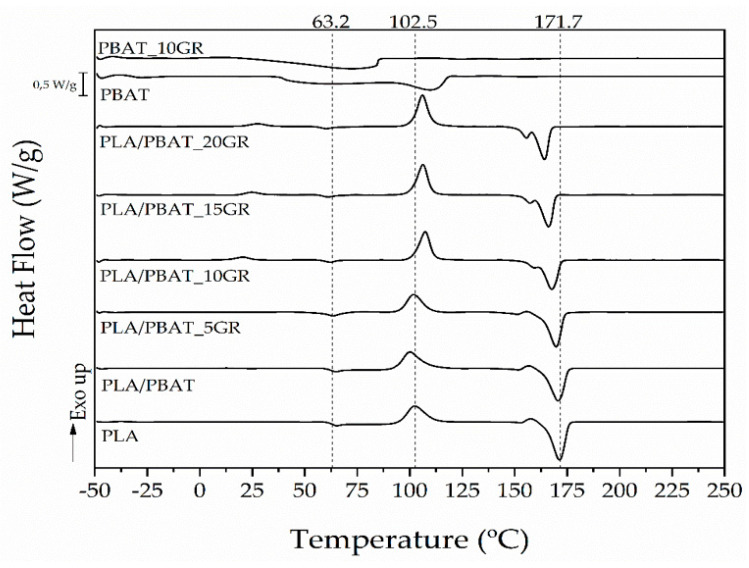
DSC curves of PLA, PBAT, PBAT_10GR, and the studied PLA/PBAT formulations with different content of GR.

The calorimetric curves of the PLA, PBAT, PBAT_10GR, PLA/PBAT, and PLA/PBAT formulations containing 5, 10, 15, and 20 phr of GR formulation were obtained by differential scanning calorimetry (DSC analysis) and are reported in Figure 4. In addition, Table 4 shows the main thermal transitions such as the glass transition temperature (T_g_), cold crystallization temperature (T_cc_), the meting (∆H_m_) and crystallization (∆H_cc_) enthalpies, and the degree of crystallinity (X_c_).

The T_g_ related to the PLA component of the blend tended to decrease both when adding PBAT (1 °C lower for the PLA/PBAT formulation, due to the partial miscibility between PLA and PBAT) and when adding GR resin. Neat PLA had a T_g_ value of 63.2 °C, while this value significantly dropped (*p* < 0.05) to 57.9 °C for the T_g_ of the PLA/PBAT formulation with a 20 phr of GR. This decrease in the T_g_ of PLA is due to the saturation of GR, which acts as a lubricant, facilitating the movement of the chains. However, the PBAT_10GR had a significantly higher T_g_ than the T_g_ of the neat PBAT, going from −25.9 °C to −20.8 °C. This increment in the T_g_ when GR was additivated to the PBAT could be due to an increase in PBAT crystallinity due to the presence of the GR. It is important to mention that the T_g_ values of PBAT and PBAT_10GR formulations were obtained by DMA analysis since this transition is not easily observable by DSC.

The same effect was observed with the T_m_ of PLA and PBAT when adding GR, 171.7 °C and 110.8 °C being the temperatures for neat PLA and neat PBAT, respectively. A significant reduction to 163.9 ° C for the PLA/PBAT formulation with a 20 phr of GR and up to 79.7 °C for the PBAT_10GR formulation was achieved. This decrease confirmed the plasticizing effect of the GR resin on the PBAT and the lubricating effect exerted by the saturated GR on the PLA matrix.

Table 4 also shows the degree of crystallinity (X_c_) of the PLA fraction in the blends when adding PBAT and GR. A slight increase (not statistically significant, *p >* 0.05) in X_c_ was observed when adding PBAT, since the microdomains of PBAT act as a nucleating agent due to the interaction between PLA and PBAT. When adding 10 phr of GR, a statistically (*p* < 0.05) higher increase was observed, reaching X_c_ values of 11.6%. However, as the GR content increased above 10 phr, X_c_ decreased (reaching similar values of neat PLA) since the PBAT domains coalesced thanks to the GR and fewer nucleation points were generated from the PLA spherulites.

**Figure 5:**
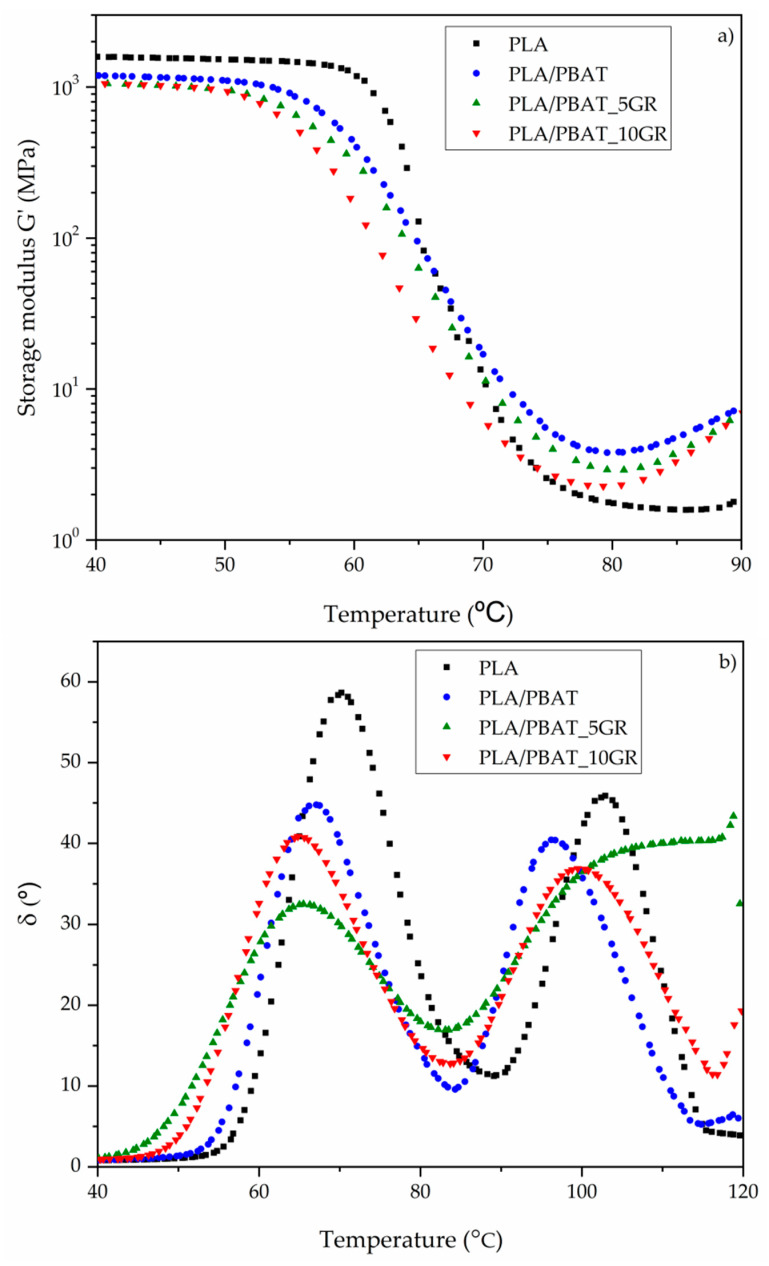
(**a**) Storage modulus (G’) and (**b**) loss factor (δ) of PLA and PLA/PBAT as references, and representatives PLA/PBAT formulations with 5 and 10 phr of GR.

By DMA technique, the T_g_ and the storage modulus “G” from torsional tests were obtained and the results are reported in Figure 5. Despite the incorporation of PBAT and GR into the PLA matrix, the values of G’ (Figure 5a) did not suffer a big change at lower temperatures, which is in good agreement with the Young’s modulus trend discussed in the mechanical characterization (Table 3). It was observed that the incorporation of 20% of PBAT generated a partially miscible blend, since T_g_ changed from 70.2 °C for neat PLA to 67.1 °C for the PLA/PBAT, as shown by the δ peaks of Figure 5b. Al-Itry et al. obtained a lower decrease (only 1 °C) in T_g_ when incorporating 20% PBAT [52]. A higher reduction in T_g_ was observed when adding GR resin. Specifically, a value of 65.5 °C was obtained for the formulation with 5 phr of GR and 64.8 °C for the formulation with 10 phr of GR. This behavior demonstrates the plasticizing effect of GR on the PBAT domains and the lubricant effect on the PLA matrix. The values of T_g_ obtained by DMA were slightly higher than those obtained by DSC but with the same trend. 

**Figure 6:**
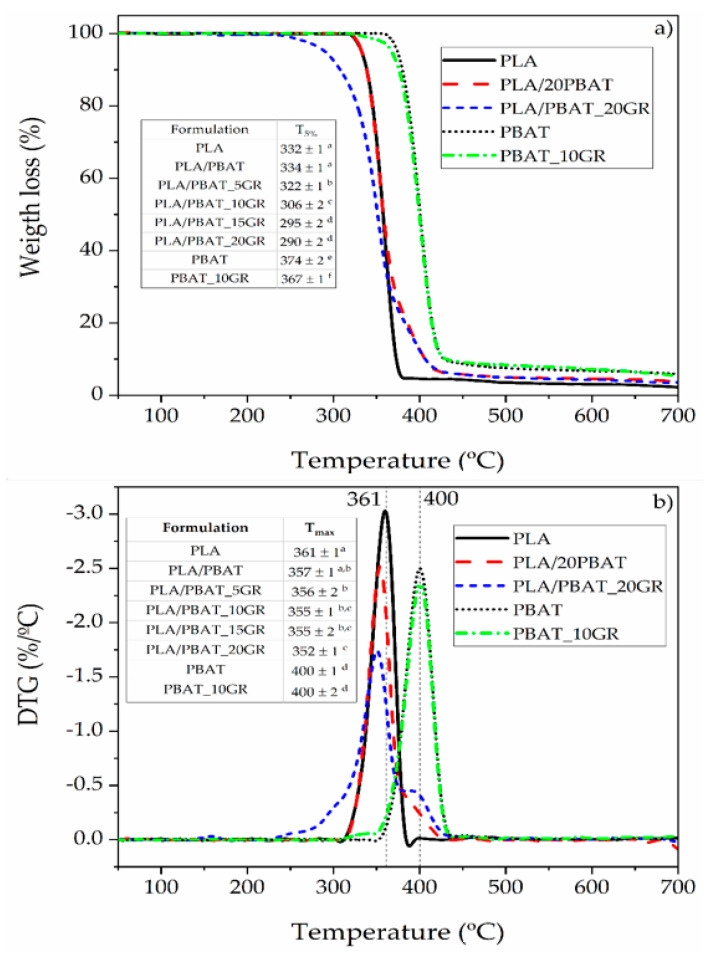
(**a**) TGA and (**b**) DTG curves of PLA and PBAT matrixes, and studied representative blends: PLA/PBAT, PLA/PBAT_20GR, and PBAT_10GR. ^a–f^ Different letters within the same property show statistically significant differences between formulations (*p* < 0.05).

Figure 6 shows the results of the thermogravimetric analysis (TGA). The T_5%_ reflects that the addition of PBAT to the PLA matrix (PLA/PBAT) did not significantly modify (*p* > 0.05) the thermal stability of neat PLA. This shows that the interactions between PLA and PBAT were poor. The T_max_ of PLA/PBAT showed a lower but not significantly different value than neat PLA (*p* > 0.05). The effect of adding GR was to obtain significantly lower T_5%_ values, which were due to the partial degradation of the GR component [38]. At higher contents of GR, the saturation of the resin directly affected the thermal stability since the components were not interacting with the PLA matrix due to the weak interaction of the PBAT domains (as also shown by FESEM).

### 3.5. Oxygen Permeability of Films of PLA/PBAT/GR Formulations 

Since these materials are intended for food packaging applications, the barrier performance against oxygen is very relevant to protect the foodstuffs from oxidation processes. Thus, the oxygen transmission rate was measured and Table 5 summarizes the OTR*e values of the obtained formulations. PLA showed significantly better oxygen barrier performance than PBAT (*p* < 0.05). The incorporation of 20 wt.% of PBAT into the formulation led to a significant increment of the oxygen permeation of PLA (increment of 72%) reaching values close to that of PBAT. This behavior was due to the low miscibility of PLA and PBAT in this formulation, which allowed oxygen diffusion through the porous/defects present in the film. The incorporation of 5 phr of GR into the PLA/PBAT blend significantly reduced the oxygen permeability by 19.5%, mainly due to the homogeneous dispersion of GR into the PLA/PBAT blend matrix. This dispersion contributed to a reduction of the defects observed in PLA/PBAT as well as to the increased crystallinity of the formulation, which led to a better oxygen barrier performance as was already observed in the PLA-based blends [19]. Higher amounts of GR, 10 phr, led to the best result, showing a significant reduction of 35% in the oxygen permeability (*p* < 0.05) with respect of PLA/PBAT formulation and being closer to that of PLA (12% higher than PLA), in good agreement with the highest crystallinity observed in this formulation. Those formulations with higher contents of GR, 15 and 20 phr, resulted in a worse oxygen barrier than PLA/PBAT_10GR but were still better than PLA/PBAT. The saturation effect of GR into the PBAT domains generated pores/defects that allowed oxygen diffusion through the film. Nevertheless, the good adhesion between the increased PBAT domains due to the GR presence with the PLA matrix at the interface still allowed to obtain better barrier performance than PLA/PBAT. The oxygen transmission results obtained here were higher than traditional petrochemical plastics widely used in the packaging sector, such as EVOH (OTR*e < 4 cm^3^mm/m^2^/day) [53] or PET (OTR*e < 3 cm^3^ mm/m^2^/day), but substantially lower than that of LDPE (OTR*e between 160 and 240 cm^3^mm/m^2^/day) [54]. Thus, the materials developed here could be used in several food packaging applications as a potential alternative to some packaging materials made of conventional plastics (i.e., polyolefins) directly as films or in more complex formulations (i.e., multilayers systems).

### 3.6. Wettability Performance of Films 

Another important issue in films for food packaging is their protection against humidity. Thus, the water contact angle was measured to get information regarding the surface hydrophilicity/hydrophobicity of the materials. Table 5 shows the water contact angle measurements. PLA presented a WCA value of 67.2°, similar to that obtained by Carbonell-Verdú et al. [22], while PBAT showed a significantly higher WCA value of 74.1°, being more hydrophobic. The PLA/PBAT blend showed even significantly higher values than those of PBAT, probably due to the high roughness of this formulation since the wettability is strongly dependent not only on the surface chemical properties but also on the surface topography [55]. The incorporation of GR into the PLA/PBAT blend produced a significant decrease (*p* < 0.05) of the WCA, particularly evident in those formulations with a high amount of GR (15 and 20 phr), increasing the hydrophilicity of the surface. Meanwhile, the incorporation of 5 and 10 phr led to a reduction of 3° of the WCA of PLA/PBAT matrix. An excess of GR, such as in the case of PLA/PBAT_15GR and PLA/PBAT_20GR, produced an increase in the size of PBAT domains. However, the WCA of these formulations were significantly lower than that of PBAT. These behaviors could be ascribed to the lower dispersion of PBAT domains into the polymeric matrix as well as the loss of the interfacial tension between PLA and PBAT [56].

## 4. Conclusions

In this work, PLA-PBAT blends were melt-blended and compatibilized through the incorporation of gum rosin. An improvement in both tensile and impact toughness was observed when adding gum rosin (GR) to the formulation composed of a PLA matrix with 20 wt.% of PBAT as a ‘soft’ component. Such increment is due to the coalescent effect of the PBAT domains into the PLA matrix due to the plasticizing effect of GR. The flexural modulus was also improved and the tensile strength increased by 80% compared to the PLA/PBAT formulation. Morphologically, it was observed that the size of the PBAT domains of 2–3 µm was optimal to reduce stress concentrations in impact conditions. Concerning neat PLA, a significant reduction of up to 8 °C of the melting temperature and up to 5.3 °C of the glass transition temperature was observed, which denotes an improvement of the processability of PLA in the blends containing PBAT and GR. Regarding the application of the obtained blends as films for packaging, PLA-PBAT-GR films were transparent with luminescence (L*) values very close to neat PLA; therefore, all the films obtained presented a very good visual appearance for the intended use. Moreover, improved barrier properties were observed as a reduction in OTR*e of up to 35% compared to the PLA/PBAT blend film and additionally showing an increase in hydrophobicity, as an increase in the water contact angle value from 67.2° for PLA to 74.5° for the PLA/PBAT_10GR film formulation was observed. Finally, the obtained results show that GR can be used as a dispersed phase size control agent to improve the toughness of PLA/PBAT formulations. The results obtained demonstrate the potential of the PLA/PBAT/GR films to be produced at an industrial level and further used in the food packaging field to replace traditional non-biodegradable plastics.

## Figures and Tables

**Table 1:** Composition and coding of PLA/PBAT/GR studied blends.

Code	PLA wt.%	PBAT wt.%	GR phr
PLA	100	-	-
PLA/PBAT	80	20	-
PLA/PBAT_5GR	80	20	5
PLA/PBAT_10GR	80	20	10
PLA/PBAT_15GR	80	20	15
PLA/PBAT_20GR	80	20	20
PBAT	-	100	-
PBAT_10GR	-	100	10

**Table 2:** Color change of the studied formulations.

	Color Change			
Formulation	L*	a*	b*	YI
PLA	41.2 ± 0.7 ^a^	−1.3 ± 0.4 ^a^	−2.7 ± 0.6 ^a^	−12.3 ± 2.4 ^a^
PLA/PBAT	87.5 ± 0.3 ^b^	−0.7 ± 0.1 ^b^	2.1 ± 0.3 ^b^	3.8 ± 0.6 ^b^
PLA/PBAT_5GR	86.2 ± 0.6 ^c^	−1.7 ± 0.1 ^c^	8.0 ± 0.8 ^c^	14.5 ± 1.5 ^c^
PLA/PBAT_10GR	82.7 ± 0.9 ^d^	−1.4 ± 0.1 ^a^	17.7 ± 1.8 ^d^	33.5 ± 3.4 ^d^
PLA/PBAT_15GR	82.4 ± 0.6 ^d^	−0.9 ± 0.3 ^b^	20.7 ± 1.2 ^e^	39.1 ± 2.1 ^e^
PLA/PBAT_20GR	79.5 ± 0.8 ^e^	−0.1 ± 0.2 ^d^	28.2 ± 1.1 ^f^	53.1 ± 2.1 ^f^
PBAT	83.7 ± 0.7 ^f^	−0.6 ± 0.2 ^b^	6.3 ± 0.5 ^g^	12.5 ± 1.1 ^c^
PBAT_10GR	76.8 ± 0.5 ^g^	1.3 ± 0.3 ^e^	20.9 ± 0.7 ^e^	43.4 ± 1.4 ^g^

^a–g^ Different letters within the same property show statistically significant differences between formulations (*p* < 0.05).

**Table 3:** Mechanical properties of PLA/PBAT blends with different contents of gum rosin (GR) as additive.

	Property	Tensile Strength (MPa)	Young’s Modulus (MPa)	Elongation at Break (%)	Flexural Strength (MPa)	Flexural Modulus (MPa)	Charpy Impact Energy (KJ/m^2^)	Hardness (Shore D)	HDT Temperature (°C)
Formulation									
PLA		65.1 ± 1.7 ^a^	2100 ± 250 ^a^	6.4 ± 1.6 ^a^	108.8 ± 8.8 ^a^	3170 ± 150 ^a,b^	* 34.6 ± 2.8 ^a^	77 ± 1 ^a^	58.0 ± 0.8 ^a^
PLA/PBAT		50.5 ± 0.5 ^b^	1680 ± 200 ^b^	16.4 ± 1.2 ^b^	74.9 ± 8.6 ^b^	2720 ± 130 ^a^	5.1 ± 1.4 ^b^	71 ± 1 ^b^	57.8 ± 0.6 ^a^
PLA/PBAT _5GR		47.3 ± 1.2 ^b^	1440 ± 200 ^b^	7.3 ± 1.4 ^a^	67.2 ± 0.8 ^b^	2510 ± 30 ^a^	8.3 ± 1.2 ^b,c^	72 ± 1 ^c^	56.6 ± 0.6 ^a,b^
PLA/PBAT _10GR		41.9 ± 0.4 ^c^	1430 ± 100 ^b^	5.2 ± 0.8 ^a,c^	48.0 ± 7.8 ^c^	2530 ± 180 ^a^	9.3 ± 0.7 ^c^	71 ± 1 ^b^	55.2 ± 0.4 ^b,c^
PLA/PBAT _15GR		38.8 ± 2.8 ^c^	1510 ± 90 ^b^	3.8 ± 0.4 ^c,d^	29.7 ± 1.1 ^d^	3400 ± 190 ^b^	10.3 ± 1.3 ^c^	74 ± 1 ^d^	54.8 ± 0.8 ^b,c^
PLA/PBAT _20GR		35.8 ± 4.5 ^c^	1790 ± 220 ^a,b^	1.7 ±0.4 ^d^	28.3 ± 3.3 ^d^	3020 ± 180 ^a,b^	6.9 ± 0.3 ^b,c^	75 ± 1 ^d^	53.8 ± 0.8 ^c^
PBAT		13.6 ± 1.4 ^d^	110 ± 40 ^c^	487 ± 70 ^e^	6.8 ± 0.5 ^e^	80 ± 10 ^c^	No break	41 ± 1 ^e^	36.8 ± 0.4 ^d^
PBAT_10GR		14.9 ± 1.7 ^d^	80 ± 10 ^c^	720 ± 15 ^f^	7.2 ± 0.8 ^e^	60 ± 10 ^c^	No break	38 ± 1 ^f^	35.6 ± 0.2 ^d^

* PLA sample tested in specimens without notch. ^a–f^ Different letters within the same property show statistically significant differences between formulations (*p* < 0.05).

**Table 4:** Thermal properties of studied formulations and neat matrixes materials.

Formulation	T_gPBAT_ * (°C)	T_gPLA_ (°C)	T_ccPLA_ (°C)	∆H_ccPLA_ (J/g)	T_mPLA_ (°C)	∆H_mPLA_ (J/g)	X_c PLA_ (%)
PLA	-	63.2 ± 1.2 ^a^	102.5 ± 0.8 ^a^	26.0 ± 1.5 ^a^	171.7 ± 0.9 ^a^	−32.8 ± 1.3 ^a^	7.4 ± 0.9 ^a^
PLA/PBAT	−33.5 ± 1.1 ^a^	62.3 ± 1.6 ^a,b^	100.8 ± 0.8 ^a^	23.9 ± 1.3 ^a^	170.3 ± 1.1 ^a^	−30.5 ± 1.6 ^a,b^	8.9 ± 0.3 ^a,b^
PLA/PBAT_5GR	−20.6 ± 0.5 ^b^	61.8 ± 1.3 ^a,b^	101.8 ± 1.5 ^a^	21.6 ± 1.5 ^b^	169.3 ± 1.3 ^a,b^	−28.8 ± 1.9 ^b^	9.5 ± 0.8 ^a,b^
PLA/PBAT_10GR	−21.3 ± 1.1 ^b,c^	60.9 ± 0.9 ^a,b,c^	107.4 ± 0.6 ^b^	23.9 ± 1.0 ^a,b^	167.5 ± 1.2 ^b,c^	−31.7 ± 1.1 ^a^	11.6 ± 1.1 ^b^
PLA/PBAT_15GR	−23.1 ± 0.9 ^c,d^	59.2 ± 0.5 ^b,c^	106.5 ± 1.3 ^b^	23.3 ± 0.6 ^a,b^	165.9 ± 0.8 ^c,d^	−29.9 ± 0.9 ^a,b^	10.4 ± 0.7 ^a,b^
PLA/PBAT_20GR	−24.2 ± 1.1 ^d^	57.9 ± 1.0 ^c^	106.0 ± 1.0 ^b^	25.1 ± 1.2 ^a^	163.9 ± 0.9 ^d^	−30.0 ± 1.0 ^a,b^	8.2 ± 1.0 ^a,b^
					T_mPBAT_ (°C)	∆H_mPBAT_ (J/g)	
PBAT	−25.9 ± 0.7 ^d^	−	−	−	110.8 ± 0.9 ^e^	−21.4 ± 0.8 ^c^	
PBAT_10GR	−20.8 ± 0.7 ^b^	−	−	−	79.7 ± 1.2 ^f^	−19.4 ± 1.2 ^c^	

* T_g_ PBAT determined by DMA analysis explained below. ^a–f^ Different letters show statistically significant differences between formulations (*p* < 0.05).

**Table 5:** Oxygen permeability measurements of PLA, PLA/PBAT, and PLA/PBAT with 5, 10, 15, and 20 phr of GR resin.

Formulation	OTR*e (cm^3^·mm/m^2^/day)	Wettability (°)
PLA	43.8 ± 2.2 ^a^	67.2 ± 2.1 ^a^
PLA/PBAT	75.2 ± 0.7 ^b^	77.6 ± 1.6 ^b^
PLA/PBAT_5GR	60.5 ± 3.7 ^c,d,e^	74.4 ± 2.6 ^c^
PLA/PBAT_10GR	49.1 ± 1.7 ^a,d^	74.5 ± 1.4 ^c^
PLA/PBAT_15GR	53.4 ± 2.6 ^c,d^	69.5 ± 4.3 ^d^
PLA/PBAT_20GR	57.6 ± 1.0 ^d^	67.1 ± 3.1 ^a^
PBAT	79.2 ± 1.9 ^b^	74.1 ± 3.0 ^c^
PBAT_10GR	66.0 ± 4.2 ^e^	82.2 ± 2.9 ^e^

^a–f^ Different letters within the same property show statistically significant differences between formulations (*p* < 0.05).

## Data Availability

The data presented in this study are available on request from the corresponding author.
